# Nature-based interventions reduce physiological stress in children with chronic illnesses: evidence from salivary biomarkers

**DOI:** 10.3389/fpubh.2025.1698278

**Published:** 2025-12-11

**Authors:** Francisco Díaz-Martínez, Miguel Felipe Sánchez-Sauco, Esteban Orenes-Piñero, Maria Jose Hernández-Vera, Francisco Robledano Aymerich, Luz Claudio, Juan Antonio Ortega-García

**Affiliations:** 1 Biomedical Research Institute of Murcia Pascual Parrilla-IMIB, Murcia, Spain; 2 Pediatric Environmental Health Speciality Unit (Pehsu), Hospital University Virgen Arrixaca, Murcia, Spain; 3 Departamento de Ecologia e Hidrologia, University of Murcia, Murcia, Spain; 4 Icahn School of Medicine at Mount Sinai Department of Environmental Medicine and Public Health, New York, NY, United States; 5 Department of Pediatrics, University of Murcia, Murcia, Spain

**Keywords:** forest environments, physiological benefits, children, scientific evidence, contact nature, salivary cortisol

## Abstract

**Introduction:**

Contact with nature is increasingly recognized as an important determinant of public health, yet limited objective evidence exists regarding its benefits for children with chronic illnesses. This study aimed to evaluate the short-term effects of a forest-based intervention on stress-related salivary biomarkers in vulnerable pediatric populations, including cancer survivors and children with neurodevelopmental disorders.

**Methods:**

A quasi-experimental design was used to assess 52 children aged 8–17 years, including both patients and their healthy siblings. Participants completed a 2.5-h guided immersion in a Mediterranean forest. Saliva samples were collected immediately before and after the intervention to measure cortisol, alpha-amylase, and immunoglobulin A levels. The study was approved by the Ethics Committee of the Hospital Clínico Universitario “Virgen de la Arrixaca” on June 29, 2021 (CEIC Code, 2021-6-10-HCUVA).

**Results:**

At the group level, significant reductions in cortisol and alpha-amylase were observed following the intervention, indicating acute stress relief. Subgroup analyses revealed notable decreases in cortisol and immunoglobulin A among children with neurodevelopmental disorders, while cancer survivors showed significant reductions in alpha-amylase.

**Discussion:**

This study provides novel biomarker-based evidence that brief exposure to natural environments can reduce physiological stress in children with chronic illnesses. These findings support the integration of accessible nature-based interventions into healthcare and community programmes to promote health, resilience, and overall wellbeing in pediatric populations.

## 1 Introduction

The relationship between nature and human health has gained increasing attention in recent decades. Historically, natural environments have been perceived as spaces for healing and restoration (1), a view now supported by a growing body of scientific evidence (2). Research shows that nature exposure benefits both physical and mental health (3, 4), including reduced stress (5), improved mood, stronger immune function (6), greater physical activity (7), and overall wellbeing (8). These effects arise from complex physiological and psychological interactions that are still being studied.

Meanwhile, biodiversity loss and ecosystem degradation driven by climate change and unsustainable development (9) pose serious public health risks. This global challenge reinforces the urgency of investigating the connections between nature and health, and of promoting the integration of green and blue spaces into our communities (10). Preserving and enhancing these spaces can be particularly impactful in urban areas, where high levels of pollution and limited access to nature exacerbate health disparities (11).

Two main frameworks explain nature's health effects: Attention Restoration Theory (ART) (12) which proposes that nature restores cognitive resources, and Stress Reduction Theory (SRT) (13) which suggests it lowers stress through physiological and psychological relaxation. These mechanisms are particularly relevant for children, who are in sensitive stages of physical, emotional, and cognitive development. Children with chronic conditions—such as those with neurodevelopmental disorders or childhood cancer survivors—often experience heightened stress, impaired immune function, and psychosocial challenges. Nature-based interventions (NBIs) may offer valuable support for improving their quality of life (14).

Beyond stress reduction, nature exposure has been associated with the prevention of chronic conditions including obesity (15), diabetes (16), cardiovascular, and respiratory diseases (17), largely through promoting healthier lifestyles (18). For children, in particular, nature contact has been linked to cognitive development (19), improved physical health (20), and better stress regulation. Living in greener environments has been associated with improved memory, academic performance (21), and immune system development through exposure to environmental microbial diversity (22). However, many children—especially those living in urban settings—are experiencing a growing “nature-deficit” (23), with potential negative consequences for both mental and physical health (24).

In recent years, salivary biomarkers such as cortisol, α-amylase, and immunoglobulin A (IgA) have been increasingly used to objectively assess the physiological impacts of NBIs (25). Cortisol reflects activation of the hypothalamic-pituitary-adrenal (HPA) axis and is a well-established marker of stress (5, 26). Salivary α-amylase, regulated by the sympathetic nervous system, responds to acute stress, while IgA plays a critical role in mucosal immunity and reflects immune function under stress (27). Together, these biomarkers provide a comprehensive picture of psychophysiological responses to environmental stimuli (28).

Despite growing evidence of the benefits of nature contact, there remains a striking gap in research focused on children and adolescents with chronic illnesses. These populations are particularly susceptible to stress and immune dysregulation. Moreover, little is known about the immediate physiological effects of brief nature exposure in these groups. To address this gap, the present quasi-experimental study evaluates the acute effects of a short nature-based intervention on salivary cortisol, α-amylase, and IgA levels in children and adolescents with neurodevelopmental disorders or a history of cancer, as well as their healthy siblings.

## 2 Material and methods

### 2.1 Participants

A total of 52 participants (30 boys and 22 girls; aged 8–17 years) were recruited through two programs of the Pediatric Environmental Health Specialty Unit of the Region of Murcia (PEHSU-Murcia):

1. “*Elijo más sano,”* focused on neurodevelopmental disorders related to prenatal exposure to legal and illegal drugs, and
2. “*PLASESCAP,”* the long-term follow-up program for childhood and adolescent cancer survivors in the Region of Murcia. Healthy siblings of the patients were also included as a comparison group.

#### 2.1.1 Inclusion criteria

Children and adolescents aged 8–17 years attending PEHSU outpatient clinics with a chronic underlying condition (cancer survivors and/or children with neurodevelopmental disorders or disabilities), and healthy siblings of these patients within the same age range. All participants resided in the Region of Murcia.

#### 2.1.2 Exclusion criteria

Children younger than 8 years or older than 17 years, insurmountable language difficulties, and lacking access to a mobile phone for communication.

### 2.2 Ethical considerations

All participants and their families received a detailed explanation of the study objectives and procedures. Written informed consent was obtained from both participants and their legal guardians. Participant data were anonymized and coded throughout the study. The study was approved by the Ethics Committee of the *Hospital Cl*í*nico Universitario “Virgen de la Arrixaca”* on June 29, 2021 (CEIC Code: 2021-6-10-HCUVA).

### 2.3 Study site

**Figure 1:**
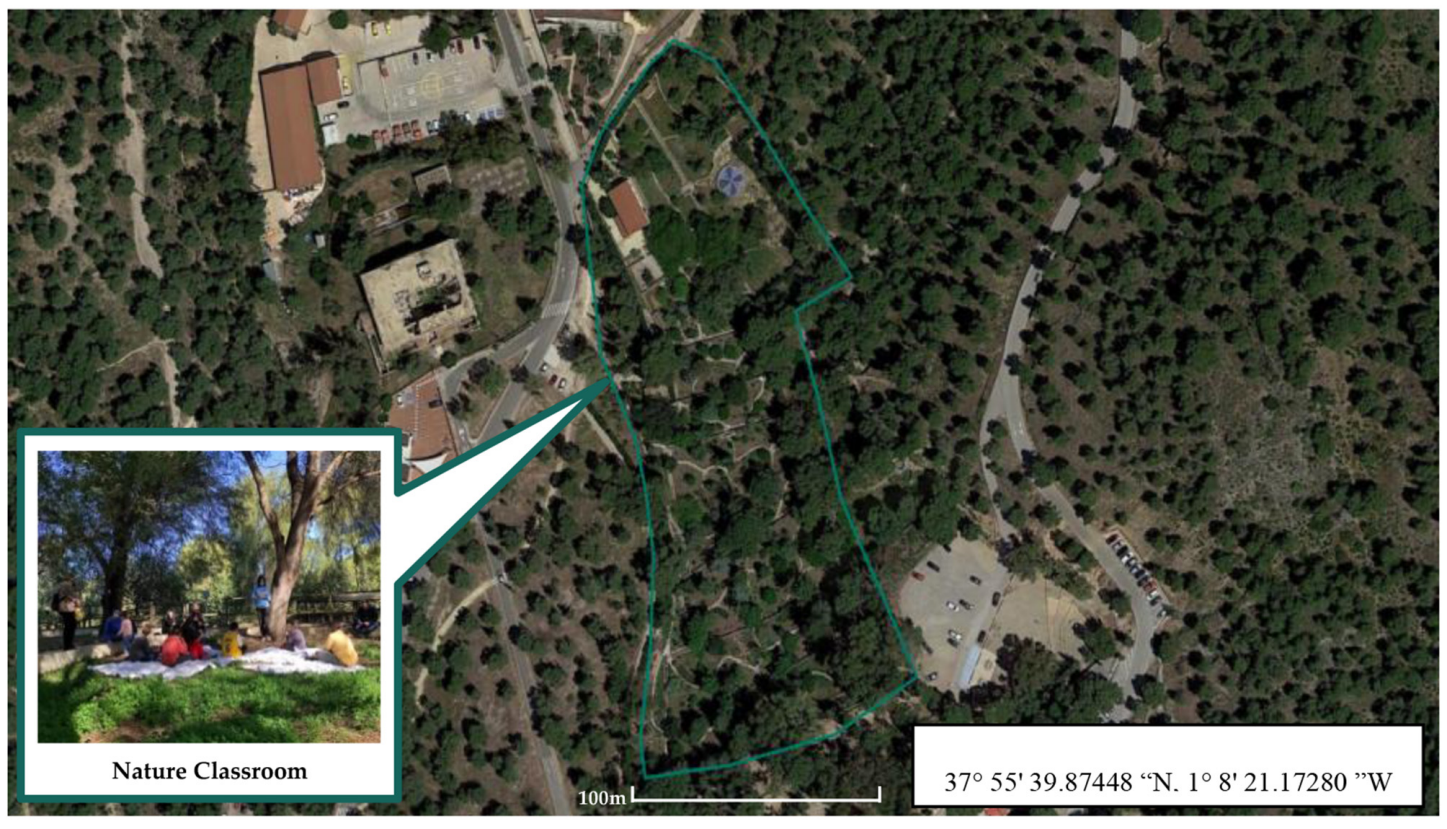
Map of the natural area (Murcia, Spain) where the intervention took place shown in Google Earth (https://earth.google.com/web/).

The study site was located in the Regional Park “El Valle y Carrascoy” (37° 5′ 39.87448″N, 1°8′ 21.17280″ W; SE Iberian Peninsula) in a typical Mediterranean forest environment with dense Aleppo pine (*Pinus halepensis*) associated with large patches of kermes oak (*Quercus rotundifolia*) and kermes oak (*Quercus coccifera*) that complete the landscape. This forest area has an altitude of 72 m above sea level. During the study period (December, 2021–February, 2023), an average maximum temperature of 20.4 °C and an average minimum temperature of 7.2°, an average monthly rainfall of 26.42 mm and an average relative humidity of 57.9% were recorded (Data obtained in 2023 from the “El Majal Blanco” Meteorological Station (37° 2′ 47.61335″ N, 1° 2′ 33.42805″ W), which depends on the State Meteorological Agency (AEMET). The park has a nature classroom and botanical garden “El Valle-Arboretum” that served as infrastructure and physical support for sampling and nature activities (Figure 1).

### 2.4 Experimental protocol

A quasi-experimental before–after design was used to assess the effects of a short nature exposure (*Educaventura*) on participants' short-term health using salivary stress biomarkers. The pre-intervention measurement (T0) served as the baseline. Before the activity, socio-environmental data, connection with nature, health-related quality of life and childhood skills and qualities were recorded using validated questionnaires.

The “*Educaventuras*” are a concept of non-pharmacological activity or intervention in nature that brings together “*educal*í*deres*” (professionals and/or volunteers trained in Environmental Health (EHS)) in carrying out activities of education and promotion of EHS with groups of children and young people. The “Educaventuras” have a duration of 2–3 h. The intervention in nature used for this study consisted of an initial walk through the forest, followed by a workshop on the senses in nature and finally a reading workshop. The average time of the activity was 3 h. The activities were led by members of PEHSU-Murcia from the University Clinical Hospital “Virgen de la Arrixaca” (HCUVA) and trained volunteers from the “Fundación Síndrome de Down” (FUNDOWN).

The study was conducted between December 11, 2021 and February 25, 2023. During this period, five activities in nature were carried out. Activities began at 10:00 a.m.

T_0_ (Baseline): anthropometric measurements, blood pressure, and heart rate were taken calmly and under standardized conditions approximately 5–10 min before starting the activity. Participants were instructed to refrain from eating or drinking before saliva collection to ensure consistent sampling conditions. Immediately afterward, saliva samples were collected (0 min) in the Nature Classroom “Arboretum-El Valle” inside the Regional Park. Participants remained seated quietly during this period to minimize potential alterations related to recent physical activity or environmental changes, ensuring basal physiological conditions for the initial cortisol sample.

Nature-based intervention: after baseline data collection, participants completed a 40-min guided forest walk, followed by a 30-min snack, a 50-min sensory workshop, and a 30-min reading activity in nature.

**Figure 2:**
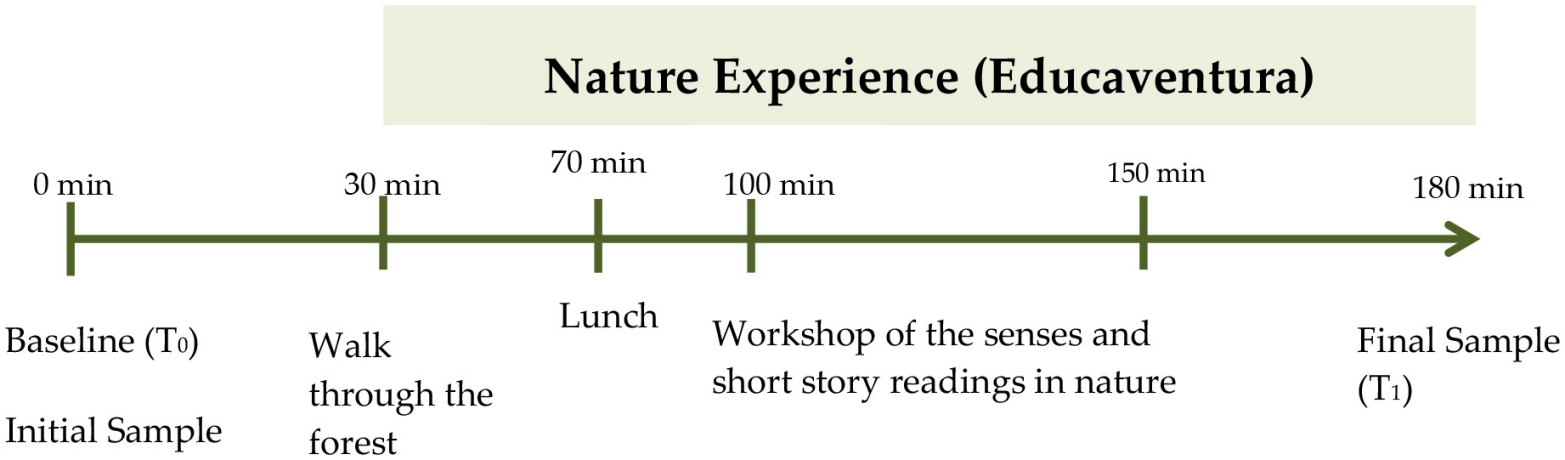
Temporal organization of the experience of the intervention in nature (*Educaventura*).

_T1_ (Post-intervention): at the end of the workshop, the final saliva collection was carried out (+2.5 h; Figure 2). The samples were kept in a freezer at −18 °C until their final storage in −60 °C freezers of the Immunology Service of the HCUVA.

### 2.5 Data collection and analysis

#### 2.5.1 Physiological indices

Saliva samples (15 ml) were collected to measure cortisol, α-amylase, and immunoglobulin A (IgA). These biomarkers are reliable, non-invasive indicators of stress in humans (29, 30). Saliva was collected twice for each patient: once in the morning before starting, and after nature exposure. Samples were immediately frozen and sent to the Proteomics Platform at IMIB “Pascual Parrilla” for analysis using commercial ELISA kits following manufacturer protocols. The kits used to measure salivary levels are the following: Human Amylase Alpha ELISA Kit from FineTest; Human IgA (Immunoglobulin A) ELISA Kit also from FineTest and Cortisol ELISA kit from Enzo.

#### 2.5.2 Anthropometric indices

Weight and height were measured before starting the activity, without shoes and with light clothing on a conventional scale and with a tape measure. The Body Mass Index (BMI) was obtained from the two previous data. Blood pressure and heart rate were evaluated in a seated position with a commercial sphygmomanometer.

#### 2.5.3 Psychological, social, and environmental indices

##### 2.5.3.1 Health-related quality of life (HRQoL)

Completed by the parents of the participating children through a telephone survey and by the children on the day of the intervention. The PedsQL 4.0 Generic Core Scales (31, 32) questionnaire was used to measure HRQOL, in its version for parents of children aged 8–12 years for the last month. This questionnaire consists of 23 items covering: (1) physical functioning (eight items), (2) emotional functioning (five items), (3) social functioning (five items); and (4) school/work functioning (five items). Each subscale is rated on a 5-point Likert-type response scale. The items are reverse-scored and linearly transformed into a scale ranging from 0 to 100. This questionnaire has demonstrated reliability and validity among the general population and has been validated in child and adolescent populations, as well as in the Spanish population. Obtaining a high score in this questionnaire was an indicator of a good quality of life in schoolchildren (Range: 0–100).

##### 2.5.3.2 Strengths and difficulties in childhood questionnaire (SDQ)

Completed by the parents of the participating children under 11 years of age, through a telephone survey. Participants aged between 12 and 17 years were self-administered. The SDQ questionnaire (The Strengths and Difficulties Questionnaire) (33, 34) consists of 25 items divided into five scales of five items each: (1) emotional symptoms scale, (2) behavioral problems scale, (3) hyperactivity scale, (4) peer problems scale; and (5) prosocial scale. The total score of difficulties is calculated by adding all the scores except those of the prosocial scale, obtaining a value of 0–40. Obtaining a high score in this questionnaire implies a higher risk of presenting mental and behavioral disorders related to neurodevelopment (with the exception of the prosocial scale; Range: 0–40).

##### 2.5.3.3 MET-h (metabolic equivalent of task per hour)

This measure quantifies energy expenditure based on the intensity and duration of physical activity. It is calculated by multiplying the METs of an activity by the time spent on it in hours. Participants were asked about the type of activities they performed and a comparative table (35) was used to calculate the MET value.

##### 2.5.3.4 Nature connection and experience index (NCEI)

Questionnaire self-completed by the participating children to assess contact with nature at home under adult supervision. It uses a scale adapted from the Child-Focused Nature Connection Index (NCI) (36, 37). It is a simple and accessible questionnaire composed of 26 items that allow an assessment of connection and contact with nature (36, 37). It is adapted to the comprehension of both children and adults, being a direct and easy to understand questionnaire, given that it uses a clear formulation of the questions accompanied by representative images. The answers are represented in the form of pictograms. Obtaining a high score in this questionnaire was an indicator of a strong connection with nature (Range: 0–40).

### 2.6 Data analysis

A total of 52 saliva samples were analyzed. Data distribution for each salivary biomarker was assessed using the Shapiro–Wilk test to verify normality. Since most variables did not meet the assumptions of normality, non-parametric analyses were conducted. The Wilcoxon signed-rank test was applied to evaluate differences between pre- and post-intervention values, and to explore potential differences across participant groups. The Mann–Whitney *U* test was used to examine sex-related differences. All statistical analyses were performed using IBM SPSS Statistics, version 24.0 (IBM Corp., Armonk, NY, USA) (38). Statistical significance was set at *p* < 0.05. Given the pilot and exploratory nature of this quasi-experimental study, the sample size was determined by feasibility and recruitment capacity rather than by an *a priori* power calculation. This approach is consistent with previous pilot studies on nature-based interventions in pediatric populations.

## 3 Results

### 3.1 General characteristics of the study population

The characteristics of the participants are shown in Table 1. A total of 52 participants (30 females and 22 males) were included in the analysis. The median age was 10 [3.50] (median [IQR]) years across both sexes. Anthropometric characteristics, including height, weight, and body mass index (BMI), were comparable between groups. No significant gender differences were observed in baseline salivary biomarker levels, health-related quality of life (PedsQL), Strengths and Difficulties Questionnaire (SDQ), or nature connectedness scores. However, boys reported a slightly higher weekly physical activity (measured in MET-h), which reached statistical significance (*p* = 0.019).

**Table 1:** General characteristics of the full sample separated by gender.

**Assessment variable**	**Sex**		***p*-value (*U* de Mann–Whitney)**	**Total (*N* = 52)**
	♀	♂		
	**(*****N*** = **30)**	**(*****N*** = **22)**		
	**Median [IQR]**	**Median [IQR]**		**Median IQR**
Age (years)	10.00 [4.00]	10.00 [3.00]	0.813	10.00 [3.00]
Height (cm)	141.50 [24.50]	140.50 [19.50]	0.911	141.00 [22.75]
Weight (kg)	35.35 [23.50]	36.25 [12.75]	0.566	35.55 [16.03]
Body mass index (BMI)	17.71 [4.88]	17.87 [3.51]	0.437	17.82 [3.45]
Cort-S pre (pg/ml)	965.51 [928.69]	1,313.50 [1,200.57]	0.322	1,262.89 [1,071.65]
Cort-S post (pg/ml)	814.98 [1,066.60]	927.89 [803.12]	0.753	869.77 [872.54]
Cort-variation	−206.66 [424.76]	−299.35 [461.93]	0.384	−221.74 [384.34]
α-Amylase pre (ng/ml)	10,258.80 [28,448.08]	18,100.25 [25,650.03]	0.890	14,112.25 [26,969.88]
α-Amylase post (ng/ml)	10,640.70 [29,222.85]	10,655.20 [30,872.08]	0.970	10,655.20 [29,879.10]
α-Amil-variation	−3,081.75 [5,756.33]	−1,145.10 [9,254.40]	0.459	−1,446.75 [7,025.48]
IgA-S pre (ng/ml)	17,280.50 [22,371.79]	14,325.00 [23,182.62]	0.993	14,920.00 [23,211.97]
IgA-S post (ng/ml)	16,496.00 [16,038.51]	13,974.35 [19,785.73]	0.882	15,291.35 [16,074.04]
IgA-variation	−1,170.02 [8,961.79]	−1,960.24 [7,435.90]	0.839	−1,511.52 [7,597.51]
PedsQL global	76.14 [37.72]	77.24 [29.78]	0.322	76.98 [28.73]
SDQ global	15.00 [13.50]	16.00 [17.25]	0.739	15.00 [17.00]
Met-h week	22.00 [16.50]	25.00 [20.30]	0.019^*^	22.30 [14.00]
NCEI	28.00 [12.50]	27.50 [11.25]	0.365	28.00 [10.00]

Cort-S pre, salivary cortisol concentration pre-intervention; Cort-S Post, salivary cortisol concentration post-intervention; α-Amylase pre, salivary α-Amylase concentration pre-intervention; α-Amylase post, salivary α-Amylase concentration post-intervention; IgA-S pre, salivary inmunoglobulin A concentration pre-intervention; IgA-S post, salivary inmunoglobulin A concentration post-intervention; PedsQL, Health-related quality of life test; SDQ, Strengths and Difficulties in Childhood Questionnaire; Met-h week, number of hours per week devoted to physical exercise; NCEI: Nature Connection and Experience Index.

The data are expressed as the median and interquartile range [IQR]. ^*^indicates statistical significance at a *p*-value <0.05.

### 3.2 Subgroups characteristics

Participants were categorized into three groups: children with neurodevelopmental disorders (*n* = 29), childhood cancer survivors (*n* = 14), and healthy siblings (*n* = 9; Table 2). No significant differences were observed between groups in terms of age, anthropometric parameters, or baseline physiological indices. BMI calculated from weight and height, is within normality for all subgroups, whose range is between 17.61 [2.96] and 18.30 [4.80]. Blood pressure and heart rate were normal at rest for the age of the participants. Health-related quality of life questionnaire (PedsQL) showed a median score of 76.98 [28.73] for the entire group, while at the subgroup level the highest score was found in cancer survivors (87.50 [26.00]) and the lowest in children with neurodevelopmental disorders due to exposure to drugs during pregnancy (73.65 [23.00]). The Qualities and Difficulties Questionnaire (SDQ) shows a score of 15 [17.00] in the whole group, the subgroup with the highest score being that of children with neurodevelopmental disorders (17 [16.00]) while that of survivors of infant-juvenile cancer presents a much lower value (7 [11.00]), showing significant statistical differences (*p* = 0.01). In the case of the NCEI, the mean value was 28 [10.00], while the highest level was obtained in survivors of childhood and adolescent cancer (28 [14.50]) and the lowest in children with neurodevelopmental disorders (27.50 [13.25]). No statistically significant differences were found in other variables. In terms of their diagnoses, all children with neurodevelopmental disorders had a range of central nervous system damage or dysfunction from exposure to alcohol and other drugs during pregnancy. In the case of cancer survivors, two had Acute Lymphoblastic Leukemia (ALL), one Acute Myeloid Leukemia (AML), two neuroblastomas, one medulloblastoma, one glioblastoma, one adrenal adenoma, one multisystem histiocytosis, one malignant neuroectodermal tumor, one astrocytoma, one Wims tumor, one Burkitt's lymphoma and one pelvic rhabdomyosarcoma. In the case of the healthy siblings subgroup, there were no known diseases.

**Table 2:** General characteristics of the separated subgroups.

**Assessment variable**	**Neurodevelopmental disorders (*N* = 29)**	**Survivors of pediatric cancer (*N* = 14)**	**Healthy siblings (*N* = 9)**	***p*-value (Kruskal–Wallis)**
	**Median [IQR]**	**Median [IQR]**	**Median [IQR]**	
Age (years)	10.00 [3.50]	10.00 [1.25]	8.00 [4.00]	0.412
Height (cm)	141.00 [21.00]	141.00 [21.00]	135.00 [26.00]	0.672
Weight (kg)	38.90 [15.25]	39.20 [15.75]	30.90 [15.13]	0.509
Body mass index (BMI)	17.61 [2.96]	18.05 [3.70]	18.30 [4.80]	0.787
Cort-S pre (pg/ml)	1,371.85 [1,230.22]	965.51 [728.34]	1,271.02 [1,514.86]	0.412
Cort-S post (pg/ml)	1,023.45 [1,212.47]	864.79 [623.29]	653.75 [1,235.40]	0.548
Cort-variation	−170.02 [583.37]	−206.66 [434.66]	−271.38 [219.80]	0.955
α-Amilasa pre (ng/ml)	23,102.7 [28,914.45]	14,112.25 [23,359.83]	4,557.40 [17,594.60]	0.431
α-Amilasa post (ng/ml)	18,768.00 [28,068.90]	5,729.55 [21,887.90]	2,355.90 [14,885.25]	0.102
α-Amil-Variation	−45.50 [10,892.00]	−3,962.10 [9,998.13]	−2,187.60 [4,559.45]	0.067
IgA-S pre (ng/ml)	23,330.00 [24,605.21]	10,787.50 [8,950.63]	15,211.00 [13,627.58]	0.168
IgA-S post (ng/ml)	14,038.46 [16,848.68]	11,268.59 [14,058.90]	24,185.00 [32,593.81]	0.155
IgA-variation	−2,371.79 [9,375.92]	−2,115.38 [8,403.63]	4,260.00 [19,930.47]	0.039^*^
PedsQL global	73.65 [23.00]	87.50 [26.00]	85.00 [54.00]	0.270
SDQ global	17.00 [16.00]	7.00 [11.00]	5.00 [8.00]	0.010^*^
Met-h week	23.00 [11.50]	14.00 [16.05]	22.00 [14.00]	0.218
Nature connection index	27.50 [13.25]	28.00 [14.50]	28.50 [11.75]	0.938

Cort-S pre, salivary cortisol concentration pre-intervention; Cort-S Post, salivary cortisol concentration post-intervention; α-Amylase pre, salivary α-Amylase concentration pre-intervention; α-Amylase post, salivary α-Amylase concentration post-intervention; IgA-S pre, salivary inmunoglobulin A concentration pre-intervention; IgA-S post, salivary inmunoglobulin A concentration post-intervention; PedsQL, Health-related quality of life test; SDQ, Strengths and Difficulties in Childhood Questionnaire; Met-h week, number of hours per week devoted to physical exercise.

The data are expressed as the median and interquartile range [IQR]. ^*^indicates statistical significance at a *p*-value <0.05.

### 3.3 Characteristics of salivary biomarkers

#### 3.3.1 Cortisol

**Figure 3:**
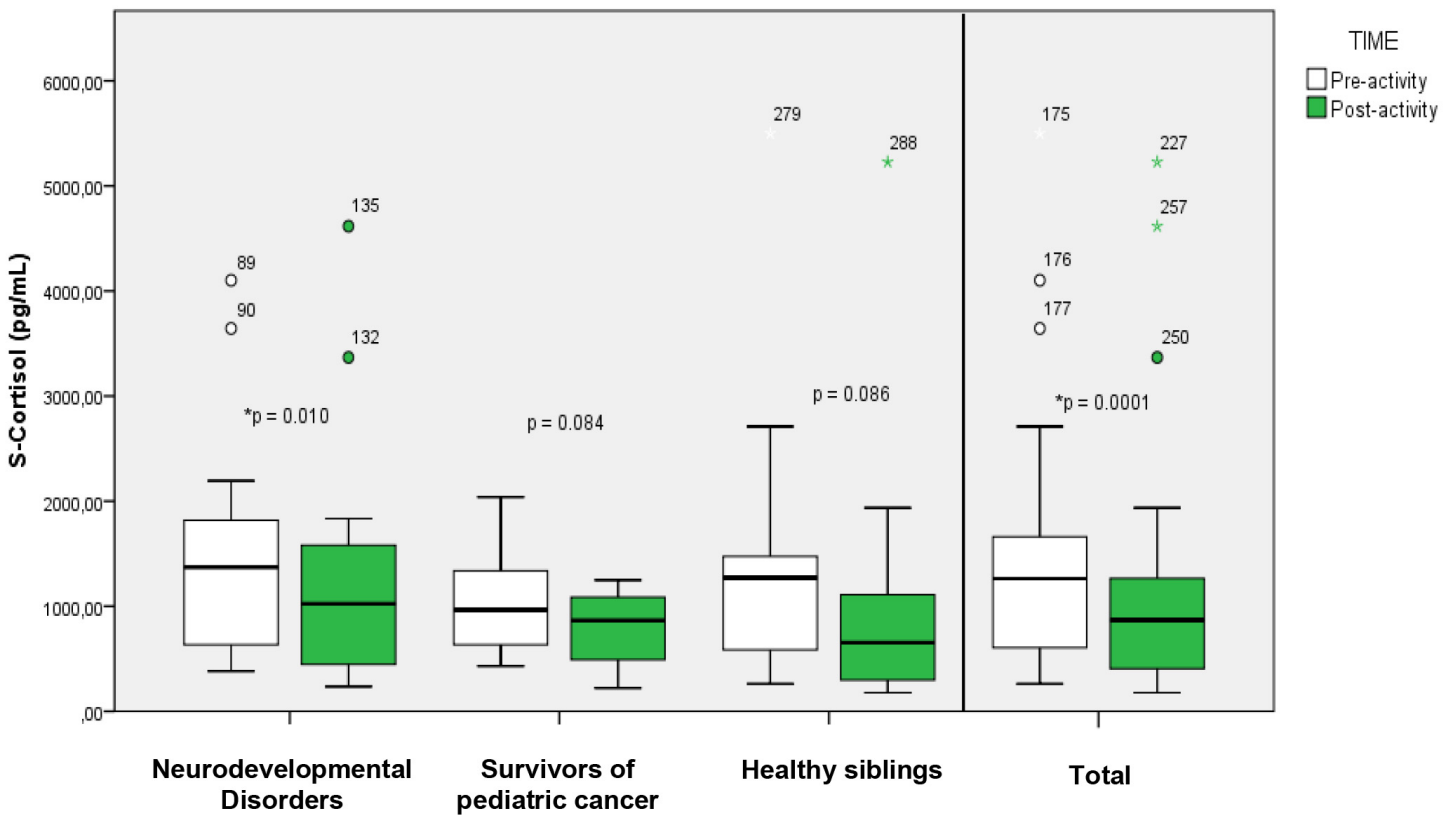
Changes (before/after) in salivary cortisol in cases. Boxes (25th−75th percentiles) show median salivary cortisol concentrations and whisker plots show minimum (5th percentiles) and maximum (95th percentiles) values. ^*^Statistically significant (*p* < 0.05).

Salivary cortisol (S-cortisol) samples show a substantial decrease after exposure in nature (Figure 3). When analyzing the results of the whole group we find a significant difference after the intervention in nature (NI) 869.77 [872.54] pg/ml (Median [IQR]); in relation to the baseline (BL; 1,262.89 [1,071.65] pg/ml). When analyzing the different subgroups separately we found statistically significant differences (*p* < 0.05) in the group of patients with neurodevelopmental disorders (BL: 1,371.85[1,230.22] pg/ml; NI: 1,023.45 [1,212.47] pg/ml). While neither in the group of cancer survivors (BL: 961.51 [728.34] pg/ml; NI: 864.79 [623.29] pg/ml) nor in the group of healthy siblings (BL: 1,271.02 [1,514.86] pg/ml; NI: 653.75 [1,235.40] pg/ml) were significant differences found, although a downward trend was observed.

#### 3.3.2 α-amylase

**Figure 4:**
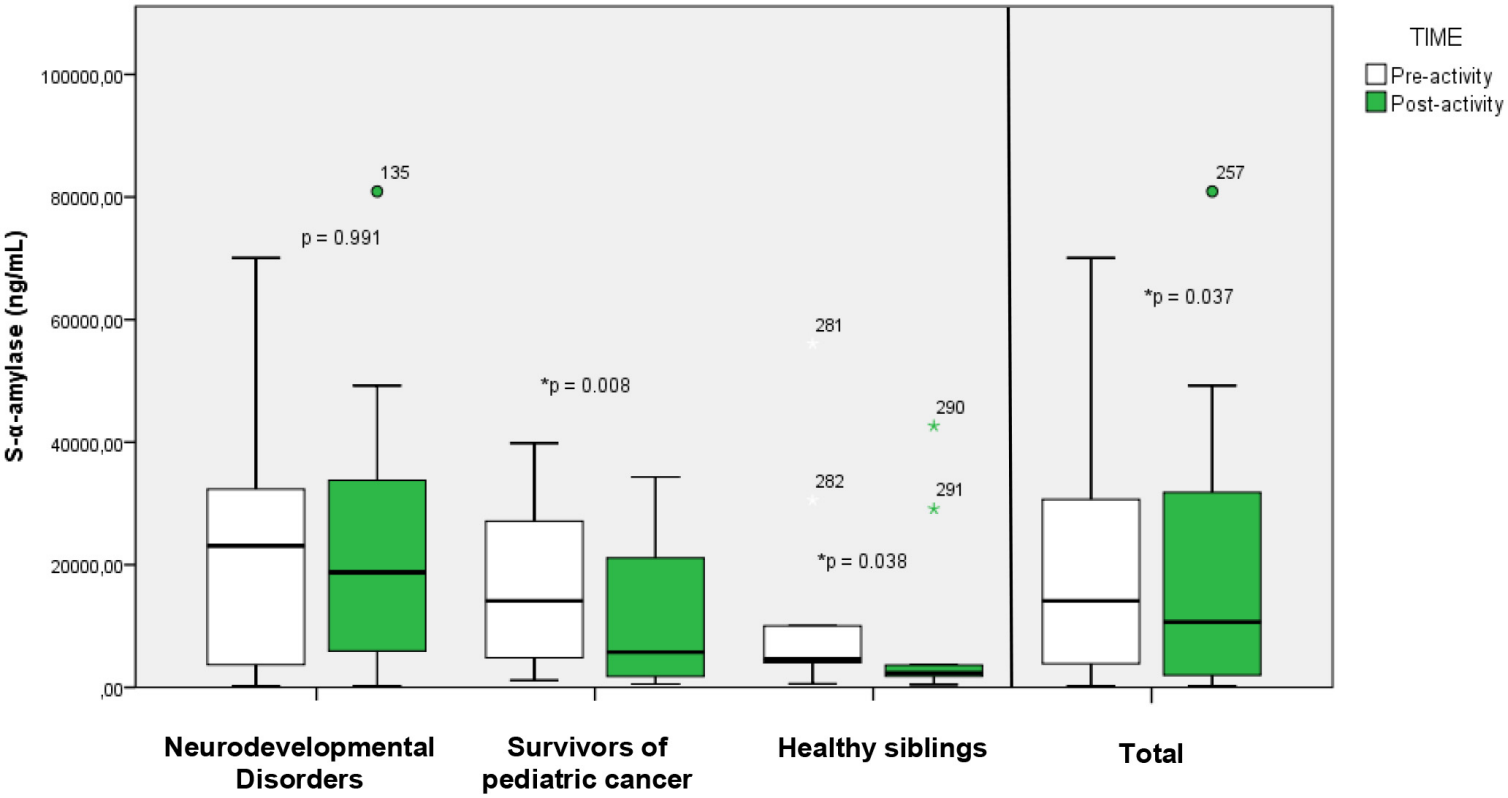
Changes (before/after) in sAA cases. Boxes (25th−75th percentiles) show median sAA concentrations and whisker plots show minimum (5th percentiles) and maximum (95th percentiles) values. *Statistically significant (*p* < 0.05).

Salivary α-amylase (S- α-amylase) samples show a similar decrease to cortisol after exposure in nature (Figure 4). When analyzing the results of the whole group we find a significant difference after the intervention in nature (NI: 10,655.20 [29,879.10] ng/ml) in relation to baseline (BL: 14,112.25 [26,969.88] ng/ml). When analyzing the different subgroups separately, we found significant differences (*p* < 0.05) in the subgroup of childhood-juvenile cancer survivors (BL: 14,112.25 [23,359.83] ng/ml; NI: 5,729.55 [21,887.90] ng/ml), and in the group of healthy siblings (BL: 4,557.4 [17,594.60] ng/ml; NI: 2,355.9 [14,885.25] ng/ml). In the neurodevelopmental disorders group, a decrease in α-amylase levels after experience in nature (BL: 23,102.7 [28,914.45] ng/ml; NI: 18,768 [28,068.90] ng/ml) is seen, although it does not reach statistical significance.

#### 3.3.3 Immunoglobulin A

**Figure 5:**
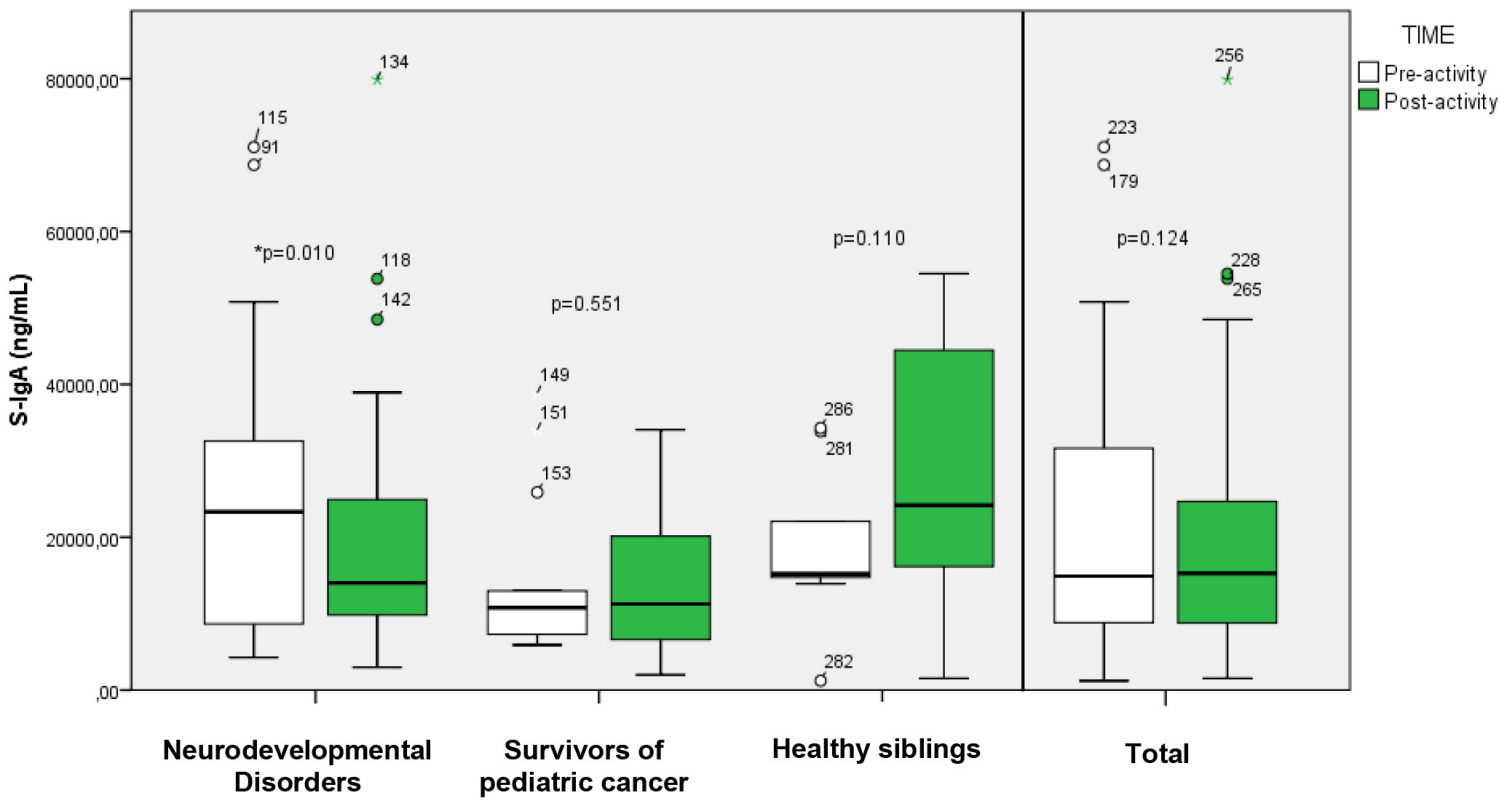
Changes (before/after) in S-IgA cases. Boxes (25th−75th percentiles) show median S-IgA concentrations and whisker plots show minimum (5th percentiles) and maximum (95th percentiles) values. ^*^Statistically significant (*p* < 0.05).

Salivary immunoglobulin A (S-IgA) samples show disparate results after exposure in nature (Figure 5). When analyzing the results of the whole group, no significant differences are found after the intervention in nature (NI: 15,291.35 [16,074.04] ng/ml) in relation to baseline (BL: 14,920.00 [23,211.97] ng/ml). When analyzing the different subgroups separately, patients with neurodevelopmental disorders (BL: 23,330 [24,605.21] ng/ml; NI: 14,038.46 [16,848.68] ng/ml) showed a statistically significant decrease. On the other hand, childhood-juvenile cancer survivors (BL: 10,787.5 [8,950.63] ng/ml; NI: 11,268.59 [14,058.90] ng/ml), and “healthy siblings” (BL: 15,211 [13,627.58] ng/ml; NI: 24,185 [32,593.81] ng/ml) showed an increase.

Subsequent statistical analyses with environmental, social, psychological and nature contact variables found no significant correlations with reflected cortisol, α-amylase and salivary immunoglobulin A levels.

## 4 Discussion

This study analyzed the response of three different human salivary stress biomarkers in pediatric patients with chronic diseases during a 2.5-h exposure to a natural forest area. We found different trends depending on the stress marker, both cortisol and amylase showed a significant decrease after exposure. However, immunoglobulin-A increased in two of the subgroups, except in patients with neurodevelopmental disorders where it experienced a decrease.

Salivary cortisol, a well-established marker of hypothalamic-pituitary-adrenal (HPA) axis activity, showed a 31.1% decrease across the whole sample following the intervention. This reduction was particularly significant in children with neurodevelopmental disorders, whose cortisol levels dropped by 25.4%. These results correspond with decreases in cortisol levels during exposure to forest environments, such as those found by Hunter et al. (2019) in which they estimated using a logarithmic model of cortisol a reduction of 20% per hour as a function of nature experience (25) reflecting a value similar to that found in this study. While the natural diurnal cycle of cortisol—with a peak in the early morning followed by a gradual decline throughout the day—could partly confound the observed decrease, this effect was minimized by standardizing the timing of saliva collection across participants. The maximum cortisol peak usually occurs upon awakening, around 8:00, and after 10:00 it usually stabilizes, dropping to 20%−25% at 14:00 in adults (39) and up to 33% at 13:00 in children (40). Children with prenatal alcohol exposure (EPA) show higher basal cortisol levels than those without EPA (41). This may be aggravated by early adverse experiences, such as social exclusion or family difficulties, which disrupt HPA regulation and increase the risk of mental and physical health problems (42). This evidence corresponds with the results of the questionnaires (PedsQL and SDQ) that reflect worse levels of both quality of life and childhood qualities and difficulties. The results found here show that the group that benefited the most with a more pronounced decrease in mean salivary cortisol levels is that of children with neurodevelopmental disorders due to prenatal exposure to legal and illegal drugs, highlighting the importance of interventions in nature in this group of participants. Although previous studies have observed significant changes in cortisol in short-term nature exposure (43), this is one of the first studies to observe changes have been observed in pediatric patients with neurodevelopmental disorders, and with respect to other types of interventions based on physical exercise (44), which did not obtain significant differences in this group of participants, our study has managed to find significant differences between before and after despite the small sample size.

Salivary α-amylase (sAA), associated with sympathetic nervous system activity and acute stress response, also decreased significantly (by 24.5%) at the group level. The most pronounced decrease was seen in childhood cancer survivors (59.4%), followed by healthy siblings (48.3%). Although the neurodevelopmental group showed an 18.8% reduction, this change was not statistically significant. sAA undergoes a natural daily cycle different from that of salivary cortisol, with lower values in the morning increasing to a maximum at midday, almost opposite to that of cortisol (45). Only a few studies have found decreases with significant differences in sAA after nature exposure accompanied by low-intensity physical exercise (25) because more intense physical exercise seems to influence the responses of this hormone (46). The differences may reflect varying levels of physical activity during the intervention or differing baseline sympathetic tone among subgroups. Nevertheless, the overall and subgroup results show a decrease in the level of sAA, which correspond with other forms of stress reduction intervention, such as yoga, meditation or moderate physical exercise (47, 48). The most outstanding results are found in the group of survivors of childhood and adolescent cancer, and nature experiences could be a type of intervention that complements more conventional interventions based on moderate physical exercise aimed at improving cancer survival and overall survival (49). The decrease in α-amylase levels among cancer survivors points to a reduction in sympathetic nervous system activity. This is noteworthy because cancer treatments, such as chemotherapy and radiation, often lead to long-term physiological and psychological stress (50). Nature interventions through intensive programs for childhood and adolescent cancer patients are already beginning to be developed, obtaining improvements in aerobic fitness and fatigue in the group subjected to a challenge in nature compared to control groups without intervention (51).

In contrast, salivary immunoglobulin A (S-IgA) showed heterogeneous patterns. At the group level, no significant changes were detected, but subgroup analyses revealed a 39.8% decrease in the neurodevelopmental group. Increases were observed among cancer survivors and healthy siblings, though not statistically significant. IgA levels are known to follow a distinct circadian rhythm, peaking upon awakening and declining steadily throughout the day (52). This biomarker, unlike the previous ones, has a longer half-life (52), making it difficult to evaluate its relationship in real time in the face of short interventions. Studies of interventions in nature using this biomarker have found significant differences after longer exposure periods. Bach et al. (53) found significant differences in a group of 31 healthy university students after 4 h of forest exposure compared to baseline values. In contrast, Tsunetsugu et al. (54) after a 15-min walk in the forest found no significant differences and no clear trend in a group of 12 university students. The positive trend found in 2/3 of the subgroups and at the whole group level could indicate an improvement of the immune system by reducing stress which may act as a strong immunosuppressant (55), especially beneficial for survivors of childhood and juvenile cancer. Moreover, IgA is a complex biomarker reflecting both immune and psychosocial stress responses (56). The divergent trends in our study may reflect individual differences in immune resilience, recent illness, or psychological states, emphasizing the need for caution when interpreting IgA changes in short interventions.

Taken together, our findings suggest that nature-based interventions can acutely reduce physiological stress, as evidenced by decreases in cortisol and α-amylase, especially among children with neurodevelopmental disorders and childhood cancer survivors. These results may support the potential of nature exposure as a complementary strategy to improve the psychosocial and physiological wellbeing (3, 4) of chronically ill children. However, IgA may not be a reliable short-term biomarker in this context, and its role warrants further investigation.

Ultimately, these findings suggest that nature-based strategies could represent a valuable complement within pediatric healthcare, education, and rehabilitation programs. In contexts of chronic stress—whether linked to illness, socioeconomic adversity, or environmental deprivation—access to natural environments may offer a feasible and non-pharmacological means of supporting child health. Nevertheless, these conclusions should be interpreted with caution given the preliminary nature of the data. Further research is required to confirm these effects, identify optimal intervention parameters, and evaluate their long-term clinical significance.

## 5 Limitations and strengths

This study focuses on pediatric patients with chronic conditions—a particularly vulnerable population that may derive substantial benefit from nature-based interventions. By employing salivary biomarkers, the study offers objective, non-invasive insights into physiological stress responses, contributing valuable evidence to the emerging field of environmental health in children. This approach addresses a pressing public health concern while reinforcing the therapeutic potential of nature exposure for improving both mental and physical health outcomes in pediatric populations.

Nonetheless, several limitations should be acknowledged. Although healthy siblings were included as a reference group, all participants underwent the same nature-based intervention; therefore, this study did not include a non-exposed control group, which limits causal inference but still allows within-group comparisons between participants with chronic conditions and their healthy siblings, who shared similar age and lifestyle characteristics. The relatively small sample size and quasi-experimental design limit the generalizability of the findings. In addition, the baseline measurement protocol, which included taking anthropometric and blood pressure measurements immediately before the first saliva sample, could have acted as a mild stressor, influencing the initial cortisol levels. The time between these measurements and the saliva collection may not have been sufficient to ensure a stable physiological baseline. Future studies should implement a longer resting acclimation period (e.g., 20–30 min) before collecting the baseline sample to ensure a more stable physiological baseline. Furthermore, future research should incorporate larger cohorts and randomized controlled trial designs to strengthen causal inference. Evaluating the long-term effects of repeated and sustained nature exposure could provide critical information on the durability and clinical relevance of the observed benefits. Finally, the application of advanced biomarker profiling techniques—such as proteomics, metabolomics, or epigenetic analyses—may offer deeper insights into the biological mechanisms through which nature influences pediatric health.

## 6 Conclusions

This quasi-experimental before–after study demonstrated that a brief, 2.5-h exposure to a forest environment led to significant reductions in salivary cortisol and α-amylase levels among children and adolescents with neurodevelopmental disorders, childhood cancer survivors, and their healthy siblings. Subgroup analyses revealed that the most pronounced changes occurred in participants with neurodevelopmental disorders, followed by cancer survivors and the siblings group. Statistically significant changes in salivary immunoglobulin A were observed only in the neurodevelopmental subgroup.

These findings suggest that short-duration nature-based interventions can elicit measurable reductions in physiological stress markers in pediatric populations with chronic conditions. As one of the first studies to document such effects in children and adolescents facing long-term health challenges, this research suggests that of integrating natural environments into supportive care strategies. Further investigation is warranted to explore long-term outcomes, optimize intervention protocols, and advance the inclusion of nature-based approaches in clinical and environmental health practice—while simultaneously supporting efforts to preserve and promote access to natural areas.

## Data Availability

The raw data supporting the conclusions of this article will be made available by the authors, without undue reservation.
